# Altered Spontaneous Neural Activity and Functional Connectivity in Parkinson’s Disease With Subthalamic Microlesion

**DOI:** 10.3389/fnins.2021.699010

**Published:** 2021-07-20

**Authors:** Bei Luo, Yue Lu, Chang Qiu, Wenwen Dong, Chen Xue, Li Zhang, Weiguo Liu, Wenbin Zhang

**Affiliations:** ^1^Department of Functional Neurosurgery, The Affiliated Brain Hospital of Nanjing Medical University, Nanjing, China; ^2^Department of Radiology, The Affiliated Brain Hospital of Nanjing Medical University, Nanjing, China; ^3^Department of Geriatrics, The Affiliated Brain Hospital of Nanjing Medical University, Nanjing, China; ^4^Department of Neurology, The Affiliated Brain Hospital of Nanjing Medical University, Nanjing, China

**Keywords:** amplitude of low-frequency fluctuation, deep brain stimulation, functional connectivity, microlesion effect, Parkinson’s disease, resting state functional MRI, subthalamic nucleus, Parkinson’s patients

## Abstract

**Background:**

Transient improvement in motor symptoms are immediately observed in patients with Parkinson’s disease (PD) after an electrode has been implanted into the subthalamic nucleus (STN) for deep brain stimulation (DBS). This phenomenon is known as the microlesion effect (MLE). However, the underlying mechanisms of MLE is poorly understood.

**Purpose:**

We utilized resting state functional MRI (rs-fMRI) to evaluate changes in spontaneous brain activity and networks in PD patients during the microlesion period after DBS.

**Method:**

Overall, 37 PD patients and 13 gender- and age-matched healthy controls (HCs) were recruited for this study. Rs-MRI information was collected from PD patients three days before DBS and one day after DBS, whereas the HCs group was scanned once. We utilized the amplitude of low-frequency fluctuation (ALFF) method in order to analyze differences in spontaneous whole-brain activity among all subjects. Furthermore, functional connectivity (FC) was applied to investigate connections between other brain regions and brain areas with significantly different ALFF before and after surgery in PD patients.

**Result:**

Relative to the PD-Pre-DBS group, the PD-Post-DBS group had higher ALFF in the right putamen, right inferior frontal gyrus, right precentral gyrus and lower ALFF in right angular gyrus, right precuneus, right posterior cingulate gyrus (PCC), left insula, left middle temporal gyrus (MTG), bilateral middle frontal gyrus and bilateral superior frontal gyrus (dorsolateral). Functional connectivity analysis revealed that these brain regions with significantly different ALFF scores demonstrated abnormal FC, largely in the temporal, prefrontal cortices and default mode network (DMN).

**Conclusion:**

The subthalamic microlesion caused by DBS in PD was found to not only improve the activity of the basal ganglia-thalamocortical circuit, but also reduce the activity of the DMN and executive control network (ECN) related brain regions. Results from this study provide new insights into the mechanism of MLE.

## Introduction

Deep Brain Stimulation (DBS) is an operation that is based on stereotactic technology that implants electrodes into specific nerve clusters or tissue structures within the brain, and regulates neuronal activity of the target nucleus by releasing pulsed electrical signals, which can alleviate symptoms of the disease (Miocinovic et al., 2013; Holiga et al., 2015; Lozano et al., 2019; Jakobs et al., 2020). DBS has emerged as an effective surgical treatment for Parkinson’s disease (PD) (Miocinovic et al., 2013; Dayal et al., 2017; Habets et al., 2018). To date, common targets of DBS treatment of PD include the subthalamic nucleus (STN) and globus pallidus internus (GPI) (Zavala et al., 2015; Edwards et al., 2017; Thevathasan et al., 2018). Neurosurgeons often observe an interesting phenomenon associated with DBS. After electrodes have been implanted into the brain, patients that suffer from PD experience a transitory improvement in motor symptoms of several days-to-weeks before the pulse generator has been turned on. This phenomenon, known as microlesion effect (MLE), can even be observed in the operating room (Jech et al., 2012; Holiga et al., 2015). However, the exact mechanism behind MLE remains unclear (Jech et al., 2012; Singh et al., 2012; Holiga et al., 2015). Some studies have suggested that MLE is related to the output of abnormal basal ganglia caused by the destruction of cells and their fibers within the target nuclei caused by electrode implantation (DeLong, 1990; Holiga et al., 2015). Additionally, MLE has been associated with rapid leakage of neurotransmitters due to synaptic destruction, as well as postoperative edema in brain tissue around the electrodes (Jech et al., 2012; Holiga et al., 2015). The damage caused by DBS is similar to the damage induced by traditional thalamotomy (Alvarez et al., 2005), except that the damage is less (Alvarez et al., 2005). Some studies have also demonstrated that MLE is likely associated with postoperative deterioration of language fluency, as well as cognitive decline (Mikos et al., 2011; Lefaucheur et al., 2012; Costentin et al., 2019).

Currently, resting state functional MRI (rs-fMRI) has been widely utilized to study brain activity and network changes among patients with neurological and mental illness (Nathan et al., 2014; Poldrack and Farah, 2015; Tessitore et al., 2019; Ge et al., 2020). Compared to fMRI in the task state, rs-fMRI is simple (Park et al., 2020), easy to perform and highly repeatable (Smitha et al., 2019). In fact, there have been a few studies that have used rs-MRI in order to study alterations in the brain activity during microlesions (Jech et al., 2012; Holiga et al., 2015). Holiga et al. (2015) suggested that MLE associated-brainstem and cerebellar activation can compensate for damaged neurons in order to maintain relatively normal motor function during the acute phase of MLE. However, a lack of control groups undermines credibility of these results. Jech et al. (2012) believe that microlesions have a significant effect on fMRI patterns caused by simple finger movement, and that the expected activation of the mesial premotor region, the primary motor cortex and basal ganglia were observed during the tapping test. However, the effect of exercise itself on brain function was ignored.

Amplitude of low-frequency fluctuation (ALFF) and functional connectivity (FC) are frequently-used resting data processing methods that can help study brain activity and networks (İçer et al., 2020). ALFF is able to reflect the magnitude of spontaneous neural activity in the brain at rest (Harrington et al., 2017; Xia et al., 2019). FC detects the correlation between instantaneous nerve activity among different spatially independent brain regions (Aertsen et al., 1989; Pando-Naude et al., 2019).

Herein, ALFF and FC methods were utilized to analyze rs-fMRI whole brain data of the DBS microlesion period in order to study functional changes within the brain region, as well as to further understand the physiological and pathological mechanisms of MLE.

## Materials and Methods

### Participants and Clinical Assessments

Overall, 43 patients with PD and 13 gender- and age-matched healthy controls (HCs) were recruited for this study. All participants were right-handed. PD patients were diagnosed according to the criteria of the UK Parkinson’s Disease Society Brain Bank and underwent surgery after neurosurgeons rigorously assessed DBS surgical adaptation. The exclusion criteria of PD included diagnosis other neurological disorders, mental illnesses or diseases that can have an effect on the central nervous system (i.e., cerebrovascular disease, brain trauma), presence of a metal foreign body in the head that can affect image quality and contraindication of MRI examination (i.e., cardiac pacemaker implantation). The HCs did not have a history of neurological and psychiatric diseases, as well as contraindications to MRI. The Hamilton Anxiety (HAMA) (Gjerris et al., 1983) and Hamilton Depression (HAMD) (Fava et al., 1982) scales were utilized for the assessment of mental and psychological states of all subjects. The Mini-Mental State Exam (MMSE) (Folstein et al., 1975) and Montreal Cognitive Assessment (MoCA) (Nie et al., 2012) helped assess cognitive and executive function. The quality of life of PD was evaluated by the 39-item Parkinson’s Disease Questionnaire (PDQ-39) (Peto et al., 1998). Furthermore, PD patients were assess across three sessions with the Unified Parkinson’s Disease Rating Scale part-III (UPDRS-III) (Goetz et al., 2008) and MMSE: three days before DBS (24 to 72 h before DBS), one day after DBS (24 to 48 h after DBS surgery), one month after DBS, and each time during off medication and absence of neurostimulation. The data for each patient three days before DBS and one day after DBS was divided into two groups of PD-Pre-DBS group and PD-Post-DBS group, respectively. The scales and MRI data of PD patients need to be collected at least 12 h after withdrawal of anti-Parkinson drugs (off-medication), in order to reduce the influence of drugs on data collection (Elfmarková et al., 2016; Manza et al., 2018). The study was granted approval by the Ethics Committee of The Brain Hospital affiliated with Nanjing Medical University on March 1, 2016, and written informed consent was obtained from all participants (Protocol Number, 2016-KY009).

### Surgery

Herein, all PD patients underwent implantation of bilateral STN deep brain electrodes and all positions of the electrode were not adjusted during the implantation process. We utilized the cartesian stereo coordinate system in order to locate the target, which takes midpoint of the line between the anterior commissure (AC) and posterior commissure (PC) as the stereotactic origin. The STN target was located at 11∼12 mm, beside the midpoint of AC and PC, 3 mm backward, and 4 mm downward. Furthermore, we fine-tuned target coordinates based on the specific location of the nuclear cluster within the MRI image (see Supplementary Table 1). Avoiding the brain sulci and deep brain blood vessels, the implanted path arc and ring angles were determined (see Supplementary Table 1). A combination of local anesthesia and general anesthesia were utilized during this procedure. The DBS electrode (model E202, PINS) implantation was performed using local anesthesia while the pulse generator (model G102R, PINS) implantation was conducted under general anesthesia. The unified standardized DBS surgery and same target location method were adopted among all PD patients via a neurosurgeon.

### Image Acquisition

The MRI data were gathered using a 1.5 Tesla GE Medical Systems scanner (produced by GE Medical System, Milwaukee, WI) using an 8-channel head coil. All subjects were then instructed to remain still, awake and think of nothing during the scan. In order to prevent and reduce the subjects’ head movement, the supporting foam pad was utilized to fix the head, and elastic earplugs were provided to reduce interference of the machine noise. The patients were scanned on both three days before DBS and one day after DBS, while the HCs group was scanned only once. The rs-fMRI data was acquired using a gradient-recalled echo-planar imaging (GRE-EPI) sequence with repetition time (TR) of 2,000 ms, echo time (TE) of 40 ms, 28 slices, thickness of 3.0 mm with no gap, flip angle (FA) of 90°, field of view (FOV) of 240 mm × 240 mm, matrix size of 64 × 64, voxel size of 3.75 mm^3^ × 3.75 mm^3^ × 3 mm^3^, and number of total volumes = 128. The T1-weighted anatomical images were acquired using 3D magnetization-prepared rapid gradient-echo (MPRAGE) sequence with TR of 11.864 ms, TE of 4.932 ms, FA of 20°, matrix size of 256 × 256, FOV of 152 mm × 152 mm, thickness of 1.4 mm, 112 slices, and voxel size of 0.59 mm^3^ × 0.59 mm^3^ × 1.4 mm^3^.

### Data Preprocessing

Functional data was preprocessed using the rs-fMRI data processing assistant (DPABI_V4.3, Beijing, China)^1^ base on the MATLAB 2013b^2^ platform. The preprocessing steps are consistent with previous literature (Xue et al., 2019; Li K. et al., 2020; Sun et al., 2020; Wang et al., 2020). The specific steps are described below. After converting the DICOM format of resting data into the NIFTI format, the first five volumes were discarded due to scanner instability. The remaining 123 phases of rs-fMRI data were corrected for differences in acquisition time between all slices of the whole brain, as well as for head movement. Subjects were excluded if they exhibited head translation exceeding 3.0 mm or rotation exceeding 3.0 degrees in any direction. Therefore, six PD patients were excluded due to excessive head movement. Next, the obtained images were normalized to the Montreal Neurological Institute (MNI) space, resampled to a voxel size of 3 mm^3^ × 3 mm^3^ × 3 mm^3^ and then spatially smoothed with a gaussian kernel with full width at half maximum (FWHM) of 4 mm × 4 mm × 4 mm. The nuisance variables included 24 motion parameters, global signals, white matter signals, cerebrospinal fluid signals, as well as linear trends were regressed out using a general linear mode. Finally, a time-bandpass filter was carried out (0.01 Hz < f < 0.10 Hz) to eliminate the influence of high frequency physiological noise, as well as low frequency drift noise.

### ALFF and FC Analysis

ALFF calculation was carried out using the DPABI_V4.3 software. All voxels were converted from the time domain to frequency domain through the use of the Fast Fourier Transform (Luo et al., 2020). The ALFF value of each voxel was obtained by averaging the square root of the power spectrum in the range of 0.01–0.10 Hz. Finally, the ALFF value of whole brain voxels was divided by mean ALFF value of all voxels in order to obtain a standardized ALFF map.

Regions that had significant ALFF differences between before and after surgery in PD patients were identified as regions of interest (ROIs). A seed-based FC analysis was carried out through the use of Resting-State f-MRI Data Analysis Toolkit (REST_V1.8, Beijing, China)^3^ (Song et al., 2011). The average time series of each ROIs was extracted, and correlation between the ROIs and the time series of each voxel in the whole brain was calculated in order to obtain FC maps. Finally, Fisher’s z transformation (Long et al., 2016) helped normalize all FC maps in order to improve the normality of the data distribution.

### Statistical Analysis

The SPSS22 software (Chicago, IL, United States) was utilized to statistically analyze the general clinical data of PD patients and HCs using Chi-square test and two-sample *t*-test, as appropriate. *P* < 0.05 is considered a statistically significant difference. Repeated measures ANOVA test was applied in order to compare the UPDRS-III scores of PD patients across different sessions.

An analysis of covariance (ANCOVA) was carried out to assess brain areas with significant ALFF/FC differences among the three cohorts (the PD-Pre-DBS group, the PD-Post-DBS group and HCs), with age and gender as covariates using SPM12.^4^ Then, significantly different ALFF/FC brain regions among the three groups were extracted as a mask for *post hoc t*-tests. Two-sample *t* test was utilized to assess ALFF differences with age and gender as covariates between HC and PD-Pre-DBS group within this extracted ALFF mask. The differences of ALFF/FC between the PD-Pre-DBS group and the PD-Post-DBS group were analyzed through the use of paired *t*-tests with mean frame-wise displacement (FD) as covariates within the above extracted ALFF/FC mask. We applied an uncorrected voxel-level threshold *p* < 0.001 to display all results. Multiple comparisons of the family-wise error (FWE) rate with cluster *p* < 0.05 was also carried out for all results. The names of the brain regions with statistically significant differences were recorded according to anatomical automatic labeling (AAL) partitioning template (Rolls et al., 2015).

## Results

### Demographic and Clinical Features

Overall, 37 PD patients and 13 HCs were included in the study (see Table 1), after excluding six patients. There were no significant differences with regards to gender (*p* = 0.99), age (*p* = 0.76) and MMSE scores (*p* = 0.14) between PD patients and HCs. However, there were significant differences in HAMA (*P* < 0.001), HAMD (*P* < 0.001) and MoCA scores (*P* < 0.001) between PD patients and HCs. The UPDRS-III (*p* < 0.001) and MMSE (*P* = 0.004) for PD patients varied significantly over three sessions.

**TABLE 1:** Demographic and clinical data of all subjects.

	**HCs (*n* = 13) Mean ± SD**	**PD (*n* = 37) Mean ± SD**	***P*-value**
Age (years)	62.46 ± 9.59	61.46 ± 9.47	0.76^a^
Sex (male/female)	6/7	17/20	0.99^b^
MoCA score	28.77 ± 1.09	24.14 ± 4.58	<0.001^a^*
HAMA score	0.39 ± 0.51	6.43 ± 3.93	<0.001^a^*
HAMD score	0.69 ± 0.85	6.49 ± 4.13	<0.001^a^*
PDQ-39 score	NA	45.32 ± 13.44	–
MMSE score			
Three days before DBS	28.08 ± 1.75	26.78 ± 2.88	0.14^a^
One day after DBS	–	25.51 ± 3.97	
One month after DBS	–	26.62 ± 2.98	0.004^c^*
UPDRS-III score			
Three days before DBS	NA	38.19 ± 14.85	–
One day after DBS	NA	27.78 ± 11.31	–
One month after DBS	NA	38.89 ± 12.73	<0.001^c^*

*HCs, healthy controls; PD, Parkinson’s disease; MMSE, Mini-Mental State Examination; MoCA, Montreal Cognitive Assessment; HAMA, Hamilton Anxiety Scale; HAMD, Hamilton Depression Scale; UPDRS-III, the Unified Parkinson’s Disease Rating Scale part-III; PDQ-39, the 39-item Parkinson’s Disease Questionnaire; NA, not applicable; PD-Pre-DBS, three days before DBS, PD-Post-DBS, one day after DBS, each time in the off medication and absence of neurostimulation; DBS, deep brain stimulation.*

*^a^P-value was obtained by a two-sample t-test between PD and HCs groups.*

*^*b*^*P*-value was obtained by a chi-square test between PD and HCs groups.*

^c^P-value was obtained by a repeated measures ANOVA test in three sessions.

*P < 0.05.

### ALFF Alterations

**FIGURE 1:**
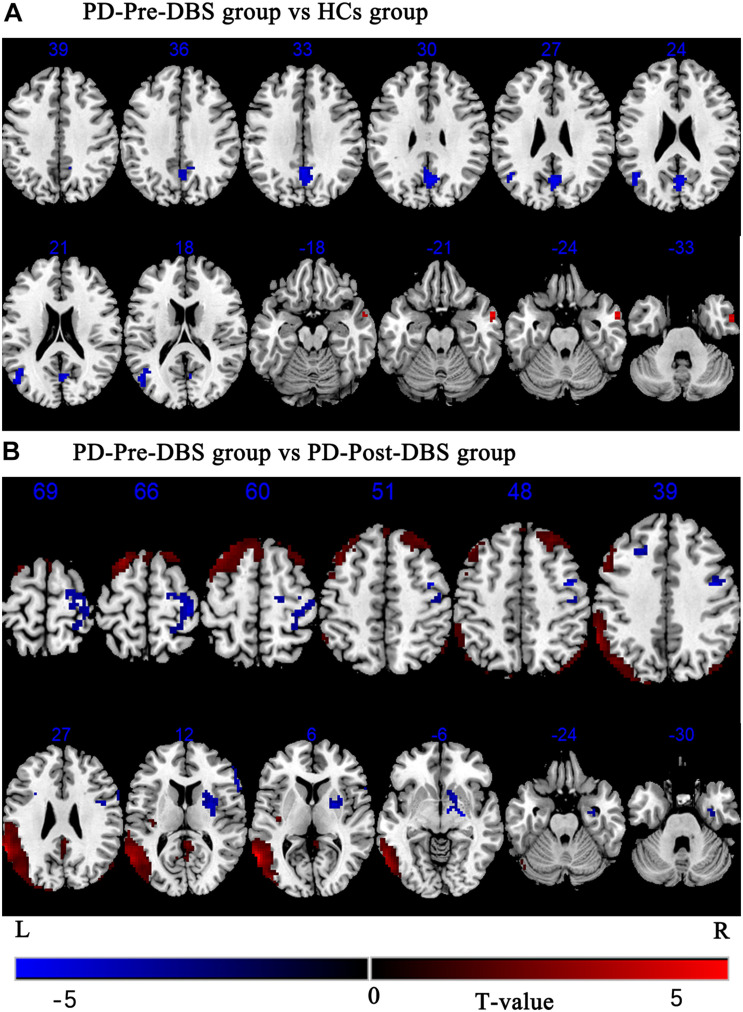
Brain regions with altered ALFF between the PD-Pre-DBS group and HCs, the PD-Pre-DBS group and the PD-Post-DBS group were shown in **(A)** (PD-Pre-DBS group vs HCs group) and **(B)** (PD-Pre-DBS group vs PD-Post-DBS group), respectively. Regions showing increased ALFF in red and decreased ALFF in blue; L and R represent left and right hemispheres, respectively; The results were corrected by multiple comparisons of the family-wise error (FWE) (voxel *p* < 0.001, cluster *p* < 0.05); ALFF, amplitude of low-frequency fluctuation; PD-Pre-DBS, three days before DBS, PD-Post-DBS, one day after DBS, each time in the off medication and absence of neurostimulation; PD, Parkinson’s disease; DBS, deep brain stimulation; vs, versus.

Compared to HCs, the PD-Pre-DBS group demonstrated increased ALFF in the right middle temporal gyrus (MTG), but decreased ALFF in the left MTG, left angular gyrus and bilateral precuneus (see Table 2 and Figure 1A).

**TABLE 2:** Alterations of ALFF between the PD-Pre-DBS group and HCs, as well as between the PD-Pre-DBS group and the PD-Post-DBS group (voxel *p* < 0.001, FWE correction with cluster *p* < 0.05).

	**Brain region (AAL)**	**Cluster size**	**Peak MNI coordinate (X, Y, Z)**			**Peak intensity**
**PD-Pre-DBS > HCs**						
Cluster1	Temporal_Mid_R	32	57	3	−21	5.4002
**HCs > PD-Pre-DBS**						
Cluster1	Temporal_Mid_L	63	−45	−60	24	−4.8854
	Angular_L					
Cluster2	Precuneus_R	122	3	−66	30	−5.0823
	Precuneus_L					
**PD-Pre-DBS > PD-Post-DBS**						
Cluster1	Temporal_Mid_L	1863	−63	−57	6	14.9574
	SupraMarginal_L					
Cluster2	Insula_L	37	−39	−15	6	6.6421
Cluster3	Precuneus_R	200	3	−42	24	7.1653
	Cingulum_Post_R					
Cluster4	Angular_R	77	36	−78	48	5.6689
Cluster5	Frontal_Mid_L	382	−39	15	57	10.5341
	Frontal_Sup_L					
Cluster6	Frontal_Sup_R	157	27	36	48	6.5654
	Frontal_Mid_R					
**PD-Post-DBS > PD-Pre-DBS**						
Cluster1	Putamen_R	223	27	0	9	−6.1208
Cluster2	Frontal_Inf_Tri_R	46	60	27	12	−4.9739
	Frontal_Inf_OperR					
Cluster3	Precentral_R	72	42	−3	48	−6.768
Cluster4	Frontal_Mid_L	48	−24	24	33	−5.4787
Cluster5	Precentral_R	191	36	−27	66	−5.7233
	Postcentral_R					

*HCs, healthy controls; PD, Parkinson’s disease; ALFF, amplitude of low-frequency fluctuation; FWE, the family-wise error; PD-Pre-DBS, three days before DBS, PD-Post-DBS, one day after DBS, one month after DBS, each time in the off medication and absence of neurostimulation; AAL, anatomical automatic labeling; MNI, Montreal Neurological Institute; DBS, deep brain stimulation.*

Relative to the PD-Pre-DBS group, the PD-Post-DBS group had higher ALFF in the right putamen, right inferior frontal gyrus (IFG) (triangular and opercular part), left middle frontal gyrus (MFG) and right precentral gyrus. However, the PD-Post-DBS group had lower ALFF in the right angular gyrus, right precuneus, right posterior cingulate gyrus (PCC), left insula, left MTG, bilateral MFG and bilateral superior frontal gyrus (dorsolateral prefrontal cortex; DLPFC) (see Table 2 and Figure 1B).

### FC Alterations

**FIGURE 2:**
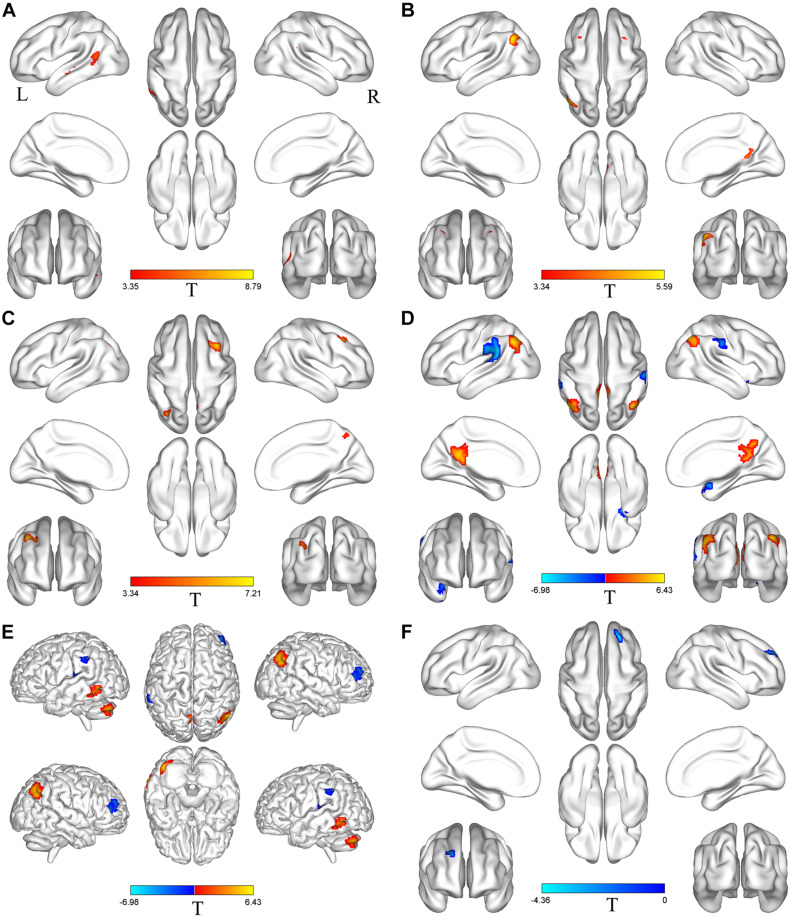
Brain regions with altered FC between the PD-Pre-DBS group and the PD-Post-DBS group. Left middle temporal gyrus **(A)**, Right precuneus **(B)**, Right angular gyrus **(C)**, Left middle frontal gyrus **(D)**, Right dorsolateral prefrontal cortex **(E)**, Right inferior frontal gyrus **(F)** as ROIs and performed a voxel-wise analysis of the whole brain FC; Regions showing increased FC in red and decreased FC in blue; L and R represent left and right hemispheres, respectively; The results were corrected by multiple comparisons of the family-wise error (FWE) (voxel *p* < 0.001, cluster *p* < 0.05); FC, functional connectivity; PD-Pre-DBS, three days before DBS, PD-Post-DBS, one day after DBS, each time in the off medication and absence of neurostimulation; PD, Parkinson’s disease; DBS, deep brain stimulation.

Based on differences between the PD-Pre-DBS and PD-Post-DBS groups, we chose left MTG, left insula, left MFG, right precuneus, right angular gyrus, right DLPFC, right putamen, right IFG and right precentral as ROIs and performed a voxel-wise analysis of the whole brain FC. There were no significant intergroup differences found in FC for three ROIs (left insula, right putamen and right precentral gyrus). Compared to the PD-Pre-DBS group, the PD-Post-DBS group demonstrated significantly decreased left MTG FC with left MTG and right superior temporal gyrus. Relative to the PD-Pre-DBS group, in right precuneus/PCC and right angular gyrus, the PD-Post-DBS group exhibited similar reduced FC in the right precuneus and bilateral MFG. Interestingly, we did not find increased FC. However, the PD-Post-DBS group had increased right IFG FC with right DLPFC. In addition, the PD-Post-DBS group had decreased left MFG FC with left precuneus and bilateral angular gyrus, but increased FC in the right temporal pole and bilateral supramarginal gyrus compared to the PD-Pre-DBS group. Furthermore, there were lower right DLPFC FC with left MTG, left cerebellum, right angular gyrus and bilateral precuneus, but higher FC with the right MTG, left superior temporal gyrus, left inferior parietal gyrus and left supramarginal gyrus in the PD-Post-DBS group than PD-Pre-DBS group. All of the above results were shown in Table 3 and Figure 2.

**TABLE 3:** Difference in functional connectivity between the PD-Pre-DBS group and the PD-Post-DBS group (voxel *p* < 0.001, FWE correction with cluster *p* < 0.05).

**Seed area**	**Brain region (AAL)**	**Cluster size**	**Peak MNI coordinate (X, Y, Z)**			**Peak intensity**
**PD-Pre-DBS > PD-Post-DBS**						
Left MTG						
Cluster 1	Temporal_Mid_L	82	−51	−42	−6	5.8543
Cluster 2	Temporal_Mid_L	268	−60	−57	21	8.7876
Cluster 3	Temporal_Sup_R	10	63	−45	21	3.9255
**Right precuneus**						
Cluster 1	Precuneus_R	51	6	−45	12	5.0775
Cluster 2	Angular_L	153	−48	−63	36	5.5852
Cluster 3	Frontal_Mid_R	25	33	21	42	4.6804
Cluster 4	Frontal_Mid_L	46	−30	24	48	4.4671
**Right angular gyrus**						
Cluster 1	Occipital_Mid_L	161	−33	−75	39	6.703
Cluster 2	Frontal_Mid_R	165	36	24	48	7.1617
Cluster 3	Frontal_Mid_L	51	−27	30	51	7.2077
Cluster 4	Precuneus_R	37	12	−60	48	5.0676
**Left MFG**						
Cluster 1	Precuneus_L	282	0	−54	24	5.2609
Cluster 2	Angular_L	217	−48	−66	36	5.8502
Cluster 3	Angular_R	130	42	−69	42	6.4341
**Right DLPFC**						
Cluster 1	Cerebelum_Crus1/2_L	57	−39	−72	−36	5.3958
Cluster 2	Temporal_Mid_L	45	−63	−54	−9	4.4681
Cluster 3	Angular_R	118	45	−69	39	5.0381
Cluster 4	Precuneus_L/R	22	3	−69	45	3.9463
**PD-Post-DBS > PD-Pre-DBS**						
**Left MFG**						
Cluster 1	Temporal_Pole_Sup_R	41	33	9	−33	−4.7855
Cluster 2	SupraMarginal_L	222	−60	−39	24	−6.9765
Cluster 3	SupraMarginal_R	36	54	−24	36	−4.2578
**Right DLPFC**						
Cluster 1	Frontal_Mid_R	49	42	48	15	−6.2616
Cluster 2	Temporal_Sup_L	20	−57	−27	18	−4.995
Cluster 3	Parietal_Inf_L	17	−60	−39	39	−4.2406
**Right IFG**						
Cluster 1	Frontal_Sup_R	23	21	48	36	−4.362

*PD-Pre-DBS, three days before DBS, PD-Post-DBS, one day after DBS, each time in the off medication and absence of neurostimulation; MTG, middle temporal gyrus; MFG, middle frontal gyrus; DLPFC, dorsolateral prefrontal cortex; IFG, inferior frontal gyrus; AAL, anatomical automatic labeling; MNI, Montreal Neurological Institute; PD, Parkinson’s disease; DBS, deep brain stimulation.*

## Discussion

Herein, we utilized ALFF combined with FC methods in order to explore changes associated with spontaneous brain activity and brain networks between HCs and PD patients before and after undergoing DBS surgery for the first time. The main focus of this study was to examine alterations of neural activity and functional networks in PD with subthalamic microlesions. After deep brain electrodes were implanted, compared to the PD-Pre-DBS group, the PD-Post-DBS group exhibited significantly increased ALFF in the basal ganglia-thalamocortical circuit (putamen, precentral gyrus), as well as decreased ALFF in the right precuneus/PCC and right angular gyrus within the default mode network (DMN) and DLPFC and MFG that belong to the executive control network (ECN). Furthermore, we observed aberrant FC in these regions with prefrontal and temporal lobes. Herein, we concluded that the brain activity and networks of PD patients were altered during the microlesion period. The results of this study complemented findings from previous studies (Jech et al., 2012; Holiga et al., 2015). On one day after surgery, the UPDRS-III scores for PD patients decreased significantly from 38.19 ± 14.85 to 27.78 ± 11.31, which is consistent with the patient’s improved postoperative symptoms. However, after one month, the scores were found to be deteriorated by 38.89 ± 12.73, which reflects a transitory characteristic of MLE (Sitburana et al., 2010; Tykocki et al., 2013). Some patients had persisted for even longer (Kondziolka and Lee, 2004). This is in line with a study that suggests that the long-term clinical effects of MLE are small (Sitburana et al., 2010). In addition, one study observed a positive correlation between MLE and the degree of motor improvement that was induced by active stimulation after surgery (Tykocki et al., 2013).

Compared to the PD-Pre-DBS group, there was significantly increased ALFF value in the right putamen of the PD-Post-DBS group. The pathophysiology of PD is characterized by a striatal dopamine deficiency due to degeneration of dopaminergic nigrostriatal neurons (Hacker et al., 2012; Huot et al., 2016; Poewe et al., 2017; Shen et al., 2020). In particular, dopamine depletion is most pronounced within the putamen (Kish et al., 1988; Goldstein et al., 2017). Additionally, the putamen is a crucial component of basal ganglia-thalamocortical circuit (Amemori et al., 2011; Morigaki and Goto, 2016), which plays a key function in the development of movement disorders in PD (DeLong and Wichmann, 2015; Morigaki and Goto, 2016; McGregor and Nelson, 2019). Many previous studies have found consistent results of reduced putamen activity in PD (Zhang et al., 2015; Wang J. et al., 2018; Hu et al., 2020). Wang J. et al. (2018) have identified a consistent decrease in putamen activity in PD using a meta-analysis and an independent validation. Zhang et al. (2015) found that tremor-dominant PD patients showed decreased regional homogeneity in right putamen compared with HCs. There are lower ALFF in the bilateral putamen compared to HCs (Hu et al., 2020). Furthermore, there was also a significantly elevated ALFF in the right precentral gyrus in PD-Post-DBS, compared to the PD-Pre-DBS group. Previous imaging studies of PD have also observed abnormalities in the precentral gyrus (Uribe et al., 2016; Li J. Y. et al., 2020). The precentral gyrus is an area that is responsible for the output of movement and a key node of the basal ganglia-thalamocortical circuit (Bradberry et al., 2012; Burciu and Vaillancourt, 2018). Some studies have discovered a decrease of regional homogeneity (Li J. Y. et al., 2020) and cortical atrophy (Huang et al., 2016; Uribe et al., 2016) within the precentral gyrus of PD. These studies even suggested that they can be used as biomarkers for PD diagnosis or prognosis (Uribe et al., 2016; Li J. Y. et al., 2020). In addition, a study of the akinetic motor subtype of PD demonstrated a decrease in gray matter volume within the motor cortex, as well as abnormal FC between these areas and the cortex involved in motor planning and execution (Kann et al., 2020). These regions may play a key role in the unique longitudinal trajectory the akinetic motor subtype of PD (Kann et al., 2020). Therefore, we thought that development of PD was related to aberrant structure and function of the motor cortex, including its neural activity (Li J. Y. et al., 2020), gray matter volume (Kann et al., 2020), cortical thickness (Huang et al., 2016) and FC (Kann et al., 2020). Herein, we hypothesized that the increased activity of PD with the subthalamic microlesion in the putamen and precentral gyrus may relatively normalize neural activity of the PD-related motor cortex and enhance functioning of the basal ganglia-thalamocortical circuit, which causes transitory improvement of patients’ symptoms after DBS. It was also helpful to understand the mechanisms of traditional thalamotomy (Alvarez et al., 2005) by studying the different causes of MLE.

Relative to the PD-Pre-DBS group, the PD-Post-DBS group had lower ALFF in the right precuneus/PCC and the right angular gyrus. These brain regions are all key components of the DMN (Seghier, 2013; Cunningham et al., 2017). Numerous studies had demonstrated that DMN has a vital function in cognitive processing of neurodegenerative diseases (Lucas-Jiménez et al., 2016; Mohan et al., 2016; Kvavilashvili et al., 2020). In addition, DMN is related to the processing of emotionally-salient stimuli, working memory and consolidation of memory (Mohan et al., 2016). Some previous studies of PD patients with mild cognitive impairments have found similar results and that the dysfunction of DMN is associated with cognitive decline in PD (Hou et al., 2016; Lucas-Jiménez et al., 2016). These findings are consistent with our research results. Our observations regarding the alterations of ALFF between the PD-Pre-DBS group and HC are in line with these studies. Our results indicated reduced ALFF in the precuneus and angular gyrus in the PD-Pre-DBS group, compared to the HCs cohort. In addition, a study discovered that the function of DMN had been compromised in cognitively unimpaired patients with PD (Tessitore et al., 2012), which suggests that PD patients had an early functional disruption of the DMN in advance of any clinical evidence of cognitive impairment. Furthermore, the right precuneus/PCC and the right angular gyrus exhibited significantly reduced FC with right precuneus, left angular gyrus and bilateral MFG in the PD-Post-DBS group compared with PD-Pre-DBS group through further FC analysis. Therefore, these results indicate that reduction of neural activity of the precuneus/PCC may cause FC impairments of the DMN (Sandrone and Catani, 2013). We also observed lower ALFF values in the left insula in the PD-Post-DBS group. The insular lobe was thought to regulate DMN, as well as the fronto-parietal network (Fathy et al., 2020), and is associated with cognitive impairment in PD (Deen et al., 2011; Fathy et al., 2020). After STN-DBS, the cognitive decline on frontal executive function (Mikos et al., 2011; Lefaucheur et al., 2012), particularly verbal fluency (Okun et al., 2009; Mikos et al., 2011; Lefaucheur et al., 2012), found in many studies is considered to be due to the surgical microlesion (Mikos et al., 2011; Lefaucheur et al., 2012). Verbal fluency still declines in the off STN-DBS states, which indicates that it is caused by surgery, rather than a stimulation-induced effect (Morrison et al., 2004; Mikos et al., 2011). The abnormality of the DMN function, caused by subthalamic microlesion, may be partly responsible for a decline in cognition, especially verbal fluency (Okun et al., 2009). Nevertheless, the absence of the assessment of postoperative language function in our study has prevented us from validating our considerations.

Compared to the PD-Pre-DBS group, the PD-Post-DBS group exhibited lower activities in the bilateral MFG and DLPFC. A PD study discovered significantly decreased gray matter volume in the superior frontal gyrus and MFG, compared to HCs (Li Y. et al., 2016). DLPFC and MFG are core and key regions of the ECN (Ham et al., 2015; Taren et al., 2017), and play an important role in maintenance and regulation of top-down modulation (Seminowicz and Moayedi, 2017), as well as driving appropriate behavioral responses (O’Reilly, 2010; Sallet et al., 2013). Furthermore, DLPFC was thought to be involved in cognitive processes (Cieslik et al., 2013; Seminowicz and Moayedi, 2017), such as attention and emotional regulation (Buhle et al., 2014; Bidet-Caulet et al., 2015). Hence, we suspected that DLPFC and MFG are involved in post-surgical cognitive decline. Further FC analysis, DLPFC and MFG exhibited decreased FC with DMN-related regions, such as the angular gyrus and precuneus, which further validated our theory. Additionally, we found a significantly elevated ALFF in the right IFG in the PD-Post-DBS group, when compared to the PD-Pre-DBS group. In some studies, this area was widely considered to have an important role in executive control function (Hampshire et al., 2010; Wang Z. et al., 2018). This finding is consistent with that of one previous study on PD patients with mild cognitive impairment that demonstrated higher ALFF in the right IFG (opercular part), which was also negatively correlated to the MoCA score (Wang Z. et al., 2018). At the same time, the PD-Post-DBS group had a significantly higher right IFG FC with right DLPFC, compared to the PD-Pre-DBS group. Therefore, we thought that hyperactivity of IFG likely indicates a compensatory effect of cognitive decline, caused by electrode implantation in PD.

FMRI has been widely utilized to study abnormal patterns of brain activity and connectivity in PD during rest and task (Wu et al., 2011; Mohl et al., 2017). In contrast to rs-fMRI, task-based fMRI requires subjects to carry out a series of specific experimental actions. Many previous studies have investigated altered brain function of the cortical and subcortical regions of the motor network in PD based on task-based MRI (Herz et al., 2014; Filippi et al., 2018). A study of Mohl et al. found that the effective connectivity of different motor networks responses to levodopa during a tapping task can distinguish the subtypes of PD (Mohl et al., 2017). In addition, PD patients exhibited decreased connectivity between the striato-cortical and striato-cerebellar pathways, while there is an elevated connectivity in the cortico-cerebellar motor areas that can possibly compensate for basal ganglia dysfunction during self-initiated movements (Wu et al., 2011). Tessa et al. discovered that PD patients have higher activity in the left primary sensorimotor cortex whose hypoactivation is associated with severity of the disease during hand motor tasks (Tessa et al., 2012). The purpose of our study is to examine changes in brain activity patterns of PD patients during microlesion stage in the resting state. Therefore, we used rs-fMRI as the imaging method in this experiment. This can help avoid a decrease in comparability of experimental results in task-based research due to different task designs and different performance of subjects. In the following study, we will use task-based fMRI in order to further explore the abnormalities of the motor network cortex in PD with subthalamic microlesions.

## Limitations

There were several limitations to our study. First, the language function were not evaluated after surgery. The main purpose of our study was to investigate the changes of motor symptoms and explore alterations of spontaneous brain activity and brain networks during the microlesion period. Therefore, we only used the UPDRS scale to evaluate the fluctuation of motor symptoms after DBS and did not evaluate the changes in speech function after DBS operation. The assessment of lack of language function did not affect the results of this study. In the following studies, we will further evaluate the cognitive and language function of patients after DBS. Secondly, although the unified standardized DBS surgery was adopted among PD patients via a neurosurgeon, we were still not able to control the absolute consistency of the position of electrodes implanted in all patients. In addition, the target implanted in this study was STN, which is divided into different sub-regions (van Wijk et al., 2020; van Wouwe et al., 2020). The damage caused by implantation in different sub-regions can induce different alterations in brain function. However, due to the small size of STN and the overlapping between functional subregions (Karachi et al., 2005), we thought that all subregions of STN were passed through by the implanted stimulation electrode (Yelnik et al., 2003), which caused minor damage to all subregions. Finally, there were much fewer HCs in our study compared to the PD group. Although the difference in sample size was a limitation of this study, there was no significant difference between demographic variables in two groups. In the future, the sample size will be expanded in order to validate results of this study.

## Conclusion

Our results demonstrate that implantation of DBS electrodes not only improves the activity of the basal ganglia-thalamocortical circuit, but also reduces activity of the DMN and ECN-related brain regions. These findings can be helpful for further understanding of potential mechanisms that underlie MLE in PD.

## Data Availability Statement

The datasets presented in this article are not readily available because the datasets analyzed in this manuscript are not publicly available. Requests to access the datasets should be directed to 18895319801@163.com.

## Ethics Statement

The studies involving human participants were reviewed and approved by the Ethics Committee of The Brain Hospital affiliated with Nanjing Medical University. The patients/participants provided their written informed consent to participate in this study.

## Author Contributions

BL and YL designed and wrote this manuscript. CQ and WD were responsible for data collection. CX and BL were responsible for data processing and analysis. LZ and WL contributed to the design of the study. WZ edited and revised the manuscript. All authors contributed to and approved the final manuscript.

## Conflict of Interest

The authors declare that the research was conducted in the absence of any commercial or financial relationships that could be construed as a potential conflict of interest.

## Footnotes

1 http://rfmri.org/dpabi

2 https://www.mathworks.com/products/matlab

3 http://restfmri.net/forum/REST_V1.8

4 https://www.fil.ion.ucl.ac.uk/spm/software/spm12/

## Abbreviations

DBS: deep brain stimulation
PD: Parkinson’s disease
STN: subthalamic nucleus
GPI: globus pallidus internus
MLE: microlesion effect
AC: anterior commissure
PC: posterior commissure
TR: repetition time
TE: echo time
FA: flip angle
IFG: inferior frontal gyrus
MFG: middle frontal gyrus
PCC: posterior cingulate gyrus
DMN: default mode network
ECN: executive control network
rs-fMRI: resting state functional MRI
ALFF: Amplitude of low-frequency fluctuation
UPDRS-III: the Unified Parkinson’s Disease Rating Scale part-III
PD-Post-DBS: one day after DBS
FC: functional connectivity
HCs: healthy controls
HAMA: Hamilton Anxiety scale
HAMD: Hamilton Depression scale
MMSE: Mini-Mental State Exam
FOV: field of view
DLPFC: dorsolateral prefrontal cortex
FWHM: full width at half maximum
ROIs: regions of interest
ANCOVA: analysis of covariance
FD: Mean frame-wise displacement
FWE: family-wise error
AAL: anatomical automatic labeling
MTG: middle temporal gyrus
MNI: Montreal Neurological Institute
MoCA: Montreal Cognitive Assessment
PDQ-39: the 39-item Parkinson’s Disease Questionnaire (PDQ-39)
PD-Pre-DBS: three days before DBS.
