# Supplementary material for: Altered Spontaneous Neural Activity and Functional Connectivity in Parkinson’s Disease With Subthalamic Microlesion
Source: Front Neurosci. 2021 Jul 20;15:699010. doi: 10.3389/fnins.2021.699010 (PMC8329380; doi:10.3389/fnins.2021.699010)
Supplement: Supplementary file 1 [file Table_1.DOCX]

**Supplementary Table 1:** The target coordinates and paths implanted into STN for all patients

| NO. | Side | X (mm) | Y (mm) | Z (mm) | Arc (mm) | Ring (mm) |
| --- | --- | --- | --- | --- | --- | --- |
| 01 | Left | 111 | 88.5 | 108 | 107 | 62 |
|  | Right | 86.5 | 88.5 | 108 | 69 | 62 |
| 02 | Left | 110.5 | 95.5 | 111 | 108 | 64 |
|  | Right | 86 | 96 | 111 | 76 | 61 |
| 03 | Left | 113 | 97 | 107.5 | 107 | 66 |
|  | Right | 90 | 96 | 107.5 | 75 | 62 |
| 04 | Left | 114 | 95 | 115 | 111 | 54 |
|  | Right | 89 | 96.5 | 115 | 75 | 61 |
| 05 | Left | 111 | 89.5 | 122 | 111 | 76 |
|  | Right | 87.5 | 90.5 | 121 | 74 | 79 |
| 06 | Left | 109.5 | 99 | 128 | 108 | 57 |
|  | Right | 87.5 | 100 | 129 | 70 | 57 |
| 07 | Left | 112.5 | 92 | 109 | 111 | 72 |
|  | Right | 89 | 93.5 | 108 | 76 | 72 |
| 08 | Left | 122 | 100.5 | 117 | 100 | 65 |
|  | Right | 82.5 | 101.5 | 115 | 82 | 65 |
| 09 | Left | 110.5 | 103 | 118 | 104 | 62 |
|  | Right | 86.5 | 103 | 118 | 70 | 61 |
| 10 | Left | 119 | 103.5 | 115 | 101 | 70 |
|  | Right | 82 | 103.5 | 113.5 | 83 | 75 |
| 11 | Left | 110.5 | 98 | 113.5 | 107 | 70 |
|  | Right | 86 | 98 | 113.5 | 76 | 67 |
| 12 | Left | 110.5 | 92.5 | 116 | 105 | 61 |
|  | Right | 87 | 93 | 116 | 69 | 63 |
| 13 | Left | 109.5 | 99.5 | 117.5 | 105 | 66 |
|  | Right | 84 | 99.5 | 117.5 | 72 | 70 |
| 14 | Left | 110 | 89.5 | 106.5 | 108 | 58 |
|  | Right | 85 | 89.5 | 106.5 | 71 | 58 |
| 15 | Left | 109.5 | 94.5 | 121.5 | 108 | 60 |
|  | Right | 89.5 | 92 | 121.5 | 73 | 58 |
| 16 | Left | 110.5 | 92.5 | 120.5 | 105 | 68 |
|  | Right | 87.5 | 92 | 122.5 | 70 | 68 |
| 17 | Left | 111 | 91.5 | 98.5 | 107 | 55 |
|  | Right | 86.5 | 92.5 | 97.5 | 74 | 54 |
| 18 | Left | 111.5 | 90 | 117.5 | 105 | 64 |
|  | Right | 86.5 | 90 | 117.5 | 73 | 65 |
| 19 | Left | 115 | 103 | 110 | 111 | 55 |
|  | Right | 93.5 | 103 | 110 | 73 | 55 |
| 20 | Left | 108.5 | 95.5 | 122 | 105 | 70 |
|  | Right | 85.5 | 95.5 | 122 | 71 | 68 |
| 21 | Left | 110 | 96 | 119.5 | 108.5 | 65 |
|  | Right | 86 | 97 | 119.5 | 68 | 65 |
| 22 | Left | 111 | 100 | 119 | 106.5 | 55 |
|  | Right | 88.5 | 100 | 119 | 72.5 | 56 |
| 23 | Left | 112 | 99.5 | 116 | 104 | 58 |
|  | Right | 88 | 100 | 116 | 74 | 58 |
| 24 | Left | 110.5 | 103.5 | 123 | 106.5 | 62 |
|  | Right | 88 | 104.5 | 123 | 73 | 62 |
| 25 | Left | 109.5 | 100.5 | 120 | 107 | 60 |
|  | Right | 84 | 100.5 | 109 | 72 | 60 |
| 26 | Left | 113 | 89.5 | 115.5 | 110 | 64 |
|  | Right | 88.5 | 89.5 | 115.5 | 73 | 61 |
| 27 | Left | 111.5 | 95 | 118.5 | 107 | 64 |
|  | Right | 88 | 94.5 | 118.5 | 75 | 66 |
| 28 | Left | 105 | 89 | 110.5 | 107 | 65 |
|  | Right | 80 | 90 | 112.5 | 70 | 64 |
| 29 | Left | 110 | 86 | 103.5 | 104 | 66 |
|  | Right | 88 | 86 | 103.5 | 70 | 68 |
| 30 | Left | 112.5 | 96 | 119 | 110 | 69 |
|  | Right | 87.5 | 95 | 118.5 | 70 | 71 |
| 31 | Left | 116 | 91 | 108 | 109 | 69 |
|  | Right | 90 | 94.5 | 108 | 71 | 67 |
| 32 | Left | 110 | 90.5 | 104 | 106 | 72 |
|  | Right | 87 | 90 | 104 | 74 | 71 |
| 33 | Left | 111 | 100.5 | 119.5 | 109 | 61 |
|  | Right | 89 | 99.5 | 119 | 70 | 63 |
| 34 | Left | 113.5 | 87.5 | 114.5 | 113 | 70 |
|  | Right | 87 | 88.5 | 114.5 | 71 | 65 |
| 35 | Left | 116 | 96 | 102 | 108 | 60 |
|  | Right | 91 | 95 | 101 | 76 | 60 |
| 36 | Left | 111 | 93 | 117.5 | 110.5 | 72 |
|  | Right | 88 | 94 | 117.5 | 70 | 69 |
| 37 | Left | 110 | 95.5 | 111.5 | 109 | 66 |
|  | Right | 85 | 97 | 112 | 69 | 63 |
